# Infectivity of recombinant Torque teno sus virus 1 in pigs coinfected with swine influenza virus

**DOI:** 10.1099/jgv.0.002264

**Published:** 2026-05-07

**Authors:** Md-Tariqul Islam, Angela Pillatzki, Brett Webb, Sheela Ramamoorthy

**Affiliations:** 1 Department of Microbiological Sciences, North Dakota State University, Fargo, ND, USA; 2 Animal Disease Research and Diagnostic Laboratory, South Dakota State University, Brookings, SD, USA; 3 Veterinary Diagnostic Laboratory, North Dakota State University, Fargo, ND, USA

**Keywords:** coinfection, ELISA, flow cytometry, influenza, qPCR, swine influenza virus (SIV), Torque teno virus (TTV)

## Abstract

Torque teno viruses (TTVs) are small non-enveloped DNA viruses that are ubiquitous among mammalian species. Although their pathogenic potential remains uncertain, TTVs can modulate host immunity and potentiate coinfecting pathogens or comorbid conditions. Progress in understanding TTV biology has been constrained by the absence of robust *in vitro* and *in vivo* experimental systems. To address these limitations, we investigated the infectivity of a recombinant swine TTV [Torque teno sus virus 1 (TTSuV1)] generated with a reverse genetics system in a snatch-farrowed piglet model. Additionally, we evaluated the impact of TTSuV1 coinfection on the pathogenesis and replication dynamics of swine influenza virus (SIV). Infection of piglets with the rescued TTSuV1 induced seroconversion and lymphopenia. Early infection was associated with reduced lymphocyte proliferative responses to mitogens and diminished or absent recall responses to viral antigens. TTSuV1 antigen was detected within immune cell populations by flow cytometry. Contrary to expectations, prior TTSuV1 infection did not significantly exacerbate SIV-associated clinical signs or pulmonary lesions. The use of a snatch-farrowed piglet model in combination with recombinant TTSuV1 derived from reverse genetics provides a controlled system for investigating TTV–host interactions. This approach represents a valuable tool for advancing mechanistic studies of TTV biology and its role in modulating coinfections.

## Introduction

Torque teno viruses (TTVs) are small, non-enveloped, circular, single-stranded DNA viruses that infect a wide range of mammalian species at high prevalence. These viruses belong to the genus Iotatorquevirus within the family *Anelloviridae*. In swine, two principal species have been identified – Torque teno sus virus 1 (TTSuV1) and Torque teno sus virus 2 – each encoding three known viral proteins and exhibiting extensive genetic variability [1]. Substantial sequence divergence among strains, coupled with multiple hosts and diverse genotypes and subtypes, underscores the remarkable genomic heterogeneity of Torque teno sus viruses (TTSuVs) [2,3]. The pathogenic potential of TTVs as primary disease agents remains uncertain, and they are generally regarded as components of the mammalian virome. Nonetheless, strong epidemiological associations have been reported between TTV infection and various disease conditions, including respiratory illness, hepatitis and autoimmune disorders in humans [4]. Moreover, TTV titres are known to increase markedly in immunocompromised individuals, making them a reliable biomarker for the degree of immunosuppression [4,5]. These findings highlight the importance of further elucidating TTV–host interactions. Additionally, TTV DNA has been detected as a contaminant in biological materials, such as vaccines, porcine-derived trypsin and pork products [6,8], emphasizing the need for continued investigation into their biology and transmission dynamics.

Experimental infection of gnotobiotic pigs with TTSuVs has been shown to induce hepatic, thymic and renal lesions. Moreover, coinfection with *TTSuV1* and either *porcine circovirus type 2* (PCV2) or *porcine reproductive and respiratory syndrome virus* (PRRSV) resulted in exacerbated clinical manifestations associated with the respective coinfecting pathogens [9,11]. These findings suggest that TTSuVs are unique among members of the *TTV* genus in exhibiting experimentally demonstrated potential for primary pathogenicity. In humans, acute respiratory infections involving influenza viruses have shown strong epidemiological associations with TTV presence [12,13]. Similarly, in swine populations, ~50% of healthy pigs are carriers of TTSuVs; however, in animals that succumb to porcine respiratory disease complex, the co-detection rate of TTSuVs alongside other respiratory pathogens – including swine influenza virus (SIV), PRRSV and PCV2 – increases to ~87% [14] markedly. Despite these associations, the influence of TTSuV1 coinfection on SIV pathogenesis has not yet been evaluated in controlled experimental pig models. Respiratory infections caused by SIVs represent a major source of economic loss in the swine industry. Morbidity rates can approach 100%, particularly in the presence of coinfections with other respiratory pathogens such as PRRSV and PCV2, which exacerbate clinical severity [15].

Understanding the mechanisms by which TTVs contribute to pathogenesis is critical for developing effective strategies to mitigate the impact of coinfecting pathogens. However, research progress has been hindered by difficulties in culturing TTVs *in vitro* and the absence of reliable animal models [4,16]. Recently, we demonstrated that supplementation of TTSuV1 cultures with the replicase protein derived from circoviruses significantly enhances TTSuV1 replication. The replicase proteins of both viral groups were functionally interchangeable [17]. This discovery enabled the establishment of an *in vitro* culture system capable of producing high titres of pure, recombinant TTSuV1 and advanced our understanding of interactions between TTSuV1 and circoviruses. Furthermore, infection of laboratory mice with the rescued TTSuV1 culture resulted in productive viral replication [18]. Our previous studies have also shown that cross-species detection of swine and human TTVs is common and that TTSuVs can infect human peripheral blood mononuclear cells (PBMCs) [19,20]. Lymphocytes and granulocytes are considered the primary sites of TTV replication, as primary lymphocyte cultures support viral replication [21,22]. Moreover, TTSuV1 infection has been shown to impair lymphocyte function [20] and downregulate antiviral gene expression [23]. Collectively, these findings suggest that TTSuVs have evolved sophisticated mechanisms to establish persistent infections and modulate host immune responses, potentially altering the host’s ability to respond to coinfections or other antigenic exposures. These observations underscore the need for reliable animal models to facilitate controlled investigations of TTV pathogenesis. Therefore, the primary objective of this study was to characterize the infectivity of recombinant TTSuV1 rescued from an infectious clone [17] in conventional snatch-farrowed piglets. A secondary objective was to assess the impact of TTSuV1 coinfection on SIV pathogenesis in pigs.

## Methods

### Virus culture

Recombinant swine TTV (TTSuV1) was rescued by transfection of porcine kidney cells (PK15N cells, 005-TDV, National Veterinary Services Laboratory, Ames, IA, USA) with a dimerized infectious clone of TTSuV1 (GenBank: KT037083.1) as described before [17]. As TTVs are non-cytolytic, the rescued virus was visualized by an immunofluorescence assay using a TTSuV1-specific antibody and quantified by the TCID_50_ method, essentially as previously described [17,23]. The rescued TTSuV1 was stored at −80 °C in aliquots until further use.

### Swine infection study

The infection study was conducted in 3-day-old male and female piglets collected by snatch farrowing. Pregnant sows at the North Dakota State University Agricultural Experimental Station high-health swine herd that is negative for all major swine pathogens were screened by TTSuV1-specific PCR and an immunofluorescence assay (IFA) [17]. Sows that were negative for TTSuV1 by PCR and IFA, and with parturition dates within 48 h of each other, were selected for the study. Parturition was induced by intramuscular injection of a commercial prostaglandin analogue (Lutalyse, Zoetis Inc., Parsippany, NJ), following the manufacturer’s instructions, on day 114 of pregnancy. Piglets were snatch-farrowed during parturition and placed in sterile containers. Newborn piglets of both sexes were transported to the South Dakota State University’s Animal Resource Wing and housed under BSL2 plus conditions. They were maintained on a bovine colostrum product (Nursemate plus 150, PBS Animal Health, Brookings, SD) for 3 days post-parturition, followed by milk replacer (Solustart II, Land O Lakes, AR) until the end of the study.

**Fig. 1.**
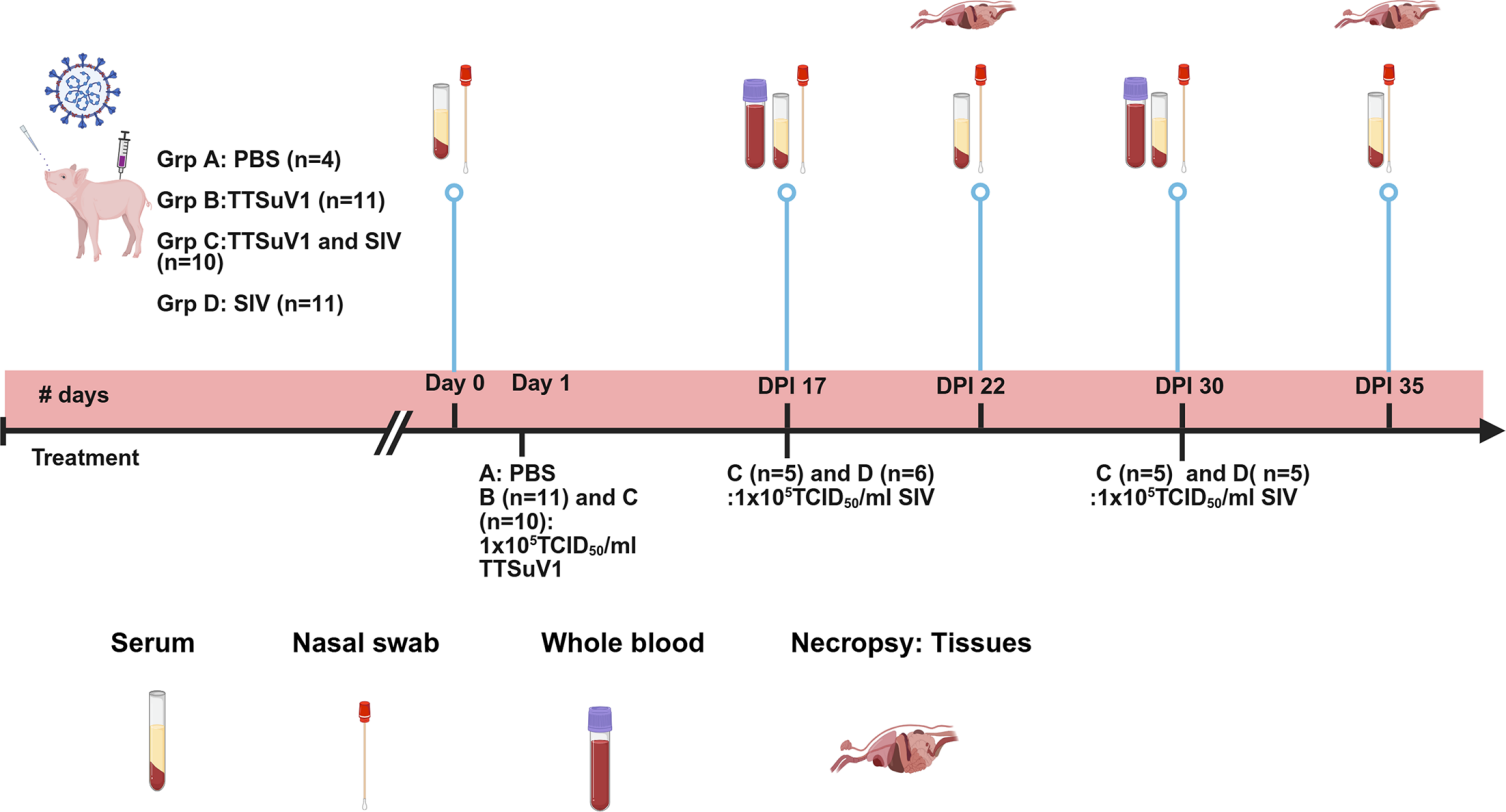
Experimental design of the swine infection model. Three-day-old male and female piglets were randomly assigned to one of four groups as follows: (A) uninfected control administered PBS (*N*=4), (B) TTSuV1 only (*N*=11), (C) TTSuV1 and SIV dual infection (*N*=10) and (D) Influenza/A/CA/2009 /H1N1 only (referred to as swine influenza virus or SIV throughout the manuscript) (*N*=11). Pigs in groups B and C were infected with TTSuV1 on DPI 1, while pigs in group A remained uninfected. To evaluate the effects of TTSuV1 coinfection on SIV infection, pigs in groups C and D were infected with SIV on DPI 17 and DPI 30. Half the pigs in each group were euthanized on DPI 22 and DPI 35 (day 5 post-SIV infection), respectively, and tissues were collected. Gross and microscopic lesions were evaluated for TTSuV1 and SIV. Serum and nasal swabs were collected for serology and qPCR on days 1, 17, 22, 30 and 35 of the study. To evaluate the effects of TTSuV1 infection on lymphocytes, whole blood was collected prior to SIV infection on DPI 17 and DPI 30. This figure was created with the BioRender software.

The piglets were randomly assigned to one of four groups as follows: (A) uninfected control administered PBS (*N*=4), (B) TTSuV1 only (*N*=11), (C) Influenza/A/CA/2009 /H1N1 only (referred to as swine influenza virus or SIV throughout the manuscript) (*N*=11) and (D) TTSuV1 and SIV coinfection (*N*=10). Pre-infection serum and nasal swabs were collected from all pigs. 1×10^6^ TCID_50_ ml^−1^ of TTSuV1 was administered to piglets in the TTSuV1 only and TTSuV1 plus SIV coinfection groups on day post-infection (DPI) 1. The dose was 2 ml intranasal and 2 ml intraperitoneal. The same volume of PBS was administered to the piglets in the uninfected group via the same routes. To evaluate the effects of SIV coinfection at two selected time points, piglets in the SIV only group and TTSuV1 plus SIV group were intra-nasally infected or coinfected, respectively, with 1×10^5^TCID_50_ ml^−1^ of Madin–Darby Canine Kidney (MDCK) cell propagated SIV culture on DPI 17 [SIV only (*N*=6), TTSuV1 plus SIV coinfection (*N*=5)] and on DPI 30 (*N*=5 in each group). The pigs in the PBS and TTSuV1 only control groups were mock-infected with PBS by the same route and dose. The doses were selected based on prior studies with SIV and porcine circovirus (PCV), which is a small DNA virus similar to TTSuV1 [24,26]. TTVs establish chronic infections, and peak viremia was achieved after 10 days post-infection in a mouse model [18]. Further, tissue lesions are clearly detected for PCV2 only after DPI 20 [24]. Therefore, DPI 17 and DPI 30 were selected as time points for the SIV coinfection. Nasal swabs and serum were collected on day 0, DPI 17, 22, 30 and 35 to assess viral replication by quantitative PCR (qPCR) and antibody responses by enzyme-linked immunosorbent assay (ELISA), respectively. Whole blood was collected on DPI 17 and DPI 30 to assess lymphocyte proliferation responses and for flow cytometry (Fig. 1). The piglets were observed daily for clinical signs due to TTSuV1 throughout the study and for SIV infection in the 6-day post-SIV infection period. Following the 6-day post-SIV infection period, piglets in each group were humanely euthanized either on DPI 22 to represent early infection [PBS (*N*=2), TTSuV1 only (*N*=6), SIV only (*N*=6), TTSuV1 plus SIV coinfection (*N*=5)] or DPI 35 [PBS (*N*=2), TTSuV1 only (*N*=5), SIV only (*N*=5), TTSuV1 and SIV coinfection (*N*=5)] to represent late infection. Following AVMA and institutional guidelines, the pigs were sedated with acepromazine (0.44 mg kg^−1^) and xylazine (0.2 mg kg^−1^), administered intramuscularly. The animals were then euthanized using a captive bolt pistol, followed by exsanguination. Major organs of the piglets were assessed for gross pathological changes during necropsy by a board-certified veterinary pathologist. Tissues were collected for microscopic examination and virus isolation. The IACUC regulations of the participating institutions were followed strictly for all animal experimentation.

### Post-infection observation

Piglets were monitored daily for respiratory distress, nasal discharge, coughing and sneezing during the study period. Body temperature measurements were obtained every other day in the post-SIV challenge period.

### Serological responses

#### TTSuV1 ELISA

**Fig. 2.**
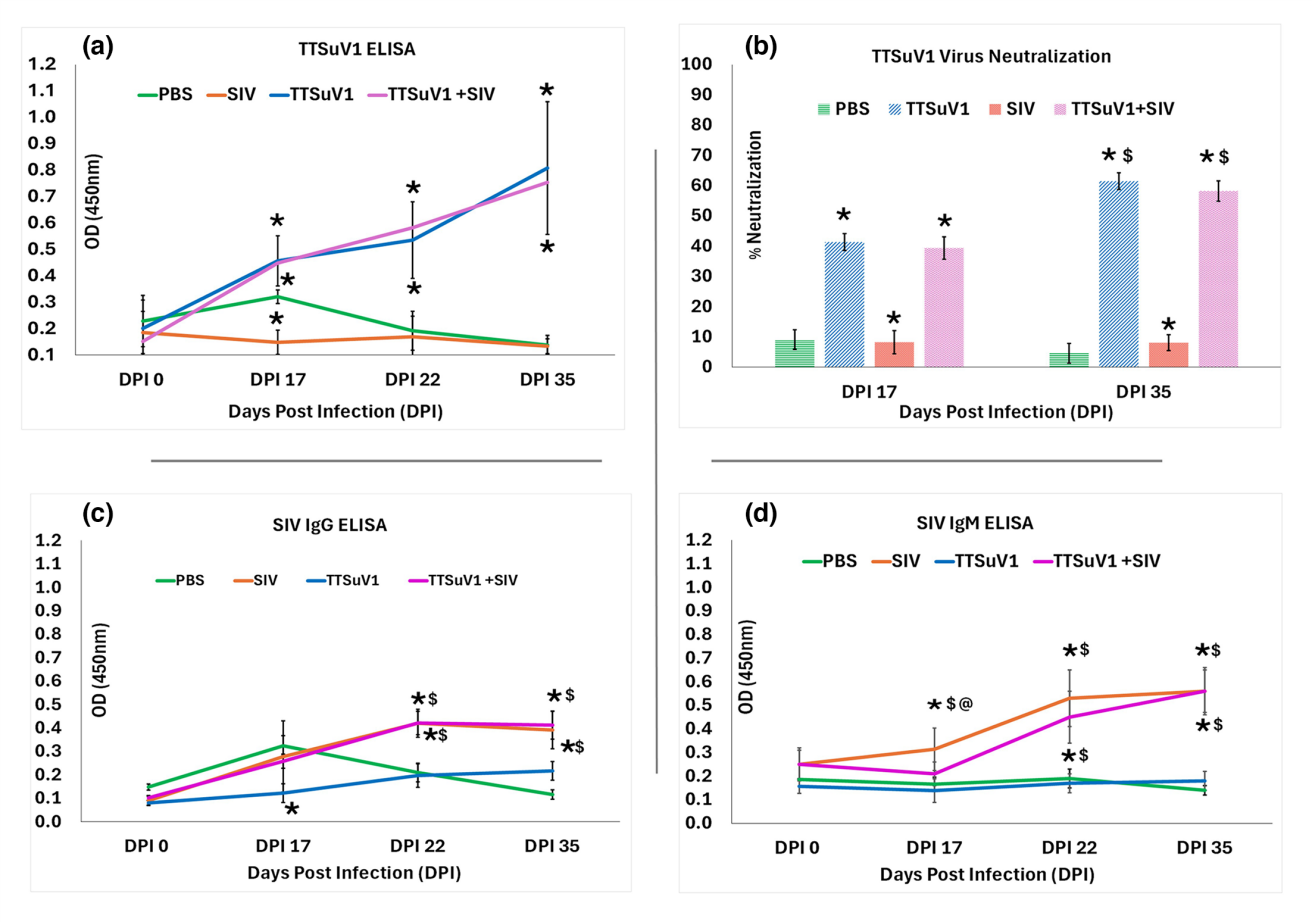
Antibody responses. (a) Anti-TTSuV1 IgG responses. A TTSuV1 ORF2-specific ELISA was used to measure antibody responses against TTSuV1. The mean values for each treatment group are depicted. *X*-axis: DPI. *Y*-axis: OD measured at 450 nm. Green, uninfected pigs; orange, SIV single infection; blue, TTSuV1 single infection; pink, TTSuV1 and Influenza/A/CA/2009 /H1N1 (SIV) coinfected pigs. *P≤0.05 when compared to the uninfected control group, Student’s t-test. Differences between the single and coinfected group were not statistically significant. (b) TTSuV1 virus neutralization responses. A fluorescent focus inhibition assay was used to measure TTSuV1-specific virus-neutralizing responses. The mean values for the test groups are depicted. *X*-axis: DPI. *Y*-axis: % virus neutralization. Green, uninfected pigs; orange, SIV single infection; blue, TTSuV1-infected pigs; pink, TTSuV1 and Influenza/A/CA/2009 /H1N1 (SIV) coinfected pigs. *P≤0.05 when compared to the uninfected control group, $*P*≤0.05 when compared to the DPI 17 data. Student’s t-test. Differences between the single and coinfected group were not statistically significant. (c) Anti-SIV IgG responses. A H1N1 N protein-specific ELISA was used to measure IgG antibody responses against SIV. The mean values for each treatment group are depicted. *X*-axis: DPI. *Y*-axis: OD measured at 450 nm. Green, uninfected pigs; orange, SIV single infection; blue, TTSuV1 single infection; pink, TTSuV1 and Influenza/A/CA/2009 /H1N1 (SIV) coinfected pigs. *P≤0.05 when compared to the uninfected control group, $*P*≤0.05 when compared to the TTSuV1 control group. Student’s t-test. Differences between the single and coinfected group were not statistically significant. (d). Anti-SIV IgM responses. A H1N1 N protein-specific ELISA was used to measure IgM antibody responses against SIV. The mean values for each treatment group are depicted. *X*-axis: DPI. *Y*-axis: OD measured at 450 nm. Green, uninfected pigs; orange, SIV single infection; blue, TTSuV1 single infection; pink, TTSuV1 and Influenza/A/CA/2009 /H1N1 (SIV) coinfected pigs. *P≤0.05 when compared to the uninfected control group, $*P*≤0.05 when compared to the TTSuV1 control group, @*P*≤0.05 when compared to the SIV control group. Student’s t-test. Differences between the single and coinfected group were not statistically significant.

Binding antibody responses to TTSuV1 were evaluated by an ORF2-specific ELISA, as described before [17,19, 20]. Sera from the experimental groups collected on DPI 0, 17, 22 and 35 were tested in duplicate in two independent assays to obtain a total of four values. Briefly, 96-well ELISA plates (High Bind Microplate, Corning^®^, Corning, NY) were coated with purified TTSuV1 ORF2 antigen (1 : 100,000 dilution) overnight, at room temperature (RT). Coated plates were blocked with General Block (Immunochemistry Technologies, Bloomington, MN) containing 2% BSA with 2% sheep serum for 1 h at 37 °C. Samples were diluted to 1 : 25 in 2% BSA+PBST and 50 µl was added to antigen-coated wells. The plates were incubated for 2 hrs at 37 °C, followed by incubation with a 1 : 2500 dilution of anti-swine HRPO-conjugate (KPL, SeraCare, Milford, MA, USA) for 45 mins. Colourimetric detection was achieved by adding 50 µl of TMB substrate (KPL, SeraCare, Milford, MA, USA) to the wells for 10 mins. The reaction was stopped with a 1M HCl solution. The plates were read at 450 nm in an ELISA plate reader (Elx800 reader, BioTek Instruments, Inc., Winooski, VT). The mean values for each group and time point are presented in Fig. 2a.

### Neutralizing antibody responses against TTSuV1

A fluorescent focus micro-neutralization assay was carried out to assess neutralizing antibody responses against TTSuV1 as described previously [27]. Sera from the PBS and TTSuV1-infected pig groups were heat-inactivated at 56 °C for 10 min and diluted to 1 : 50 in Dulbecco’s modified Eagle’s medium (DMEM, Corning Cellgro, Tewksbury, MA, USA). TTSuV1 virus was rescued as described previously [28, 17] and reconstituted to 10^3^ TCID_50_/ml. Equal volumes of the virus and 1 : 50 diluted sera each were mixed well and incubated for 1 h at 37 °C. The mixture was layered on pre-formed PK-15 cell monolayers at 50–60% confluence in triplicate and incubated for 36 h at 37 °C. A virus-only control and a media-only control were maintained to obtain baseline values. To visualize the virus by IFA, as previously described [17,20], the treated PK-15 monolayers were fixed with ice-cold acetone/methanol (1 : 1) and blocked with 2% goat serum (KPL, SeraCare, Milford, MA). They were stained with a 1 : 200 dilution of an anti-rabbit TTSuV1 antibody at 37 °C for 1 h, followed by FITC-labelled anti-rabbit IgG (1 : 100) for 45 min. The fluorescent foci were counted in a blinded fashion using a fluorescent microscope (Cytation 5, BioTek). The percentage reduction in the TTSuV1 fluorescent foci count in samples treated with sera when compared to the virus controls was calculated to obtain the mean fluorescent focus inhibition data (Fig. 2b).

### SIV ELISA

Binding antibody responses to SIV were measured as previously described [29] with some modifications. 96-well ELISA plates (High Bind Microplate, Corning^®^, Corning, NY) were coated with recombinant H1N1 NP antigen obtained from the BEI resources repository (1 : 5000 dilution) overnight, at RT. Test sera were diluted to 1 : 100 in in 2% BSA+PBST, and 50 µl was added into antigen-coated wells, after blocking for 1 h at 37 °C with General Block (Immunochemistry Technologies, Bloomington, MN) containing 2% BSA with 2% sheep serum. After a 2-h incubation at 37 °C, colourimetric detection was achieved using a 1 : 2,500 dilution of anti-swine IgG or IgM HRPO-conjugate (KPL, SeraCare, Milford, MA, USA) for 1 h, followed by 50 µl of TMB substrate (KPL, SeraCare, Milford, MA, USA) for 10 min. The reaction was stopped with a 1M HCl solution. The plates were read at 450 nm in an ELISA plate reader (Elx800 reader, BioTek Instruments, Inc., Winooski, VT). The mean values for each group and time point are presented in Fig. 2c,d.

### qPCR-based detection of TTSuV1 in pigs

**Fig. 3.**
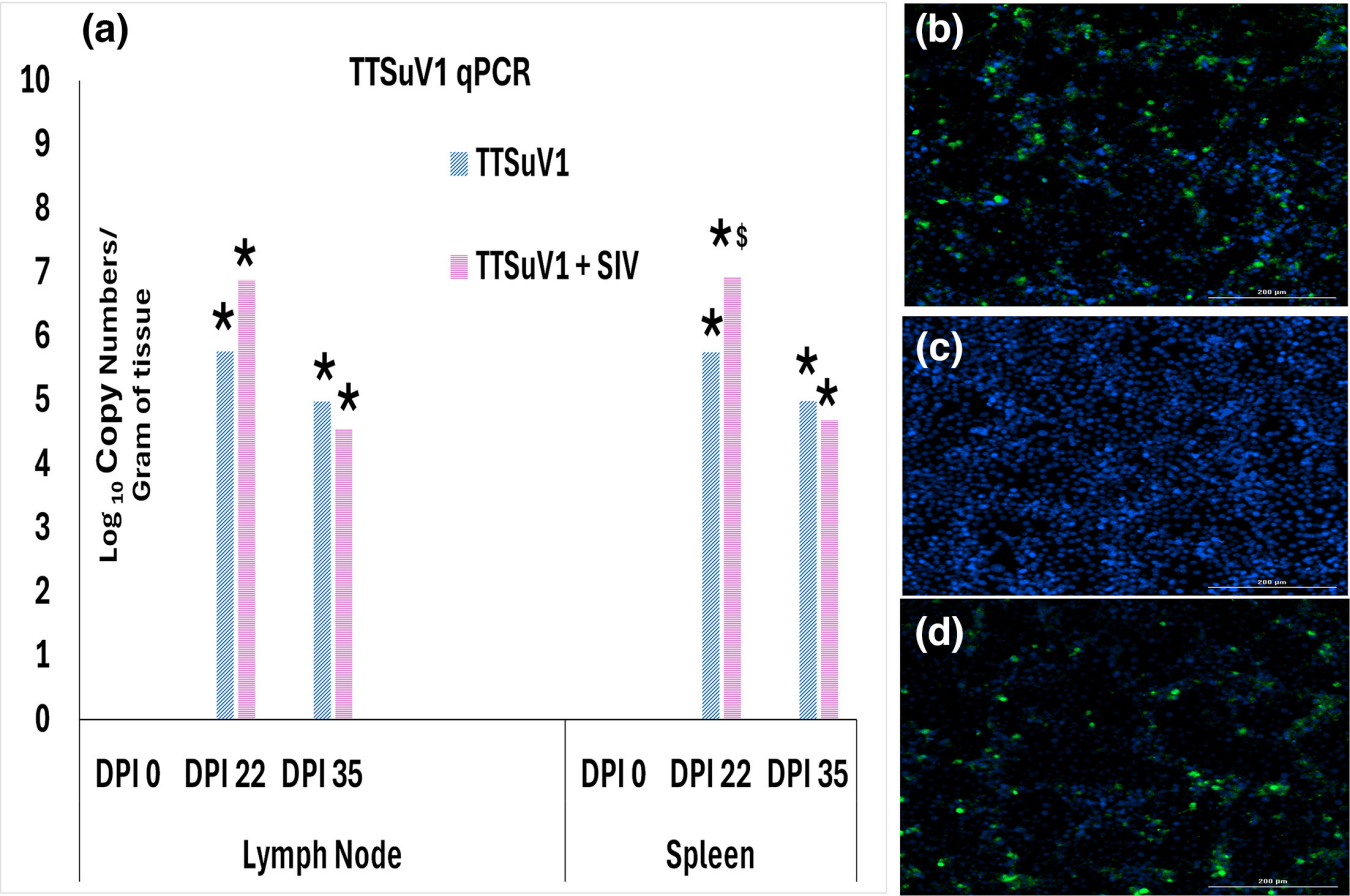
Replication of TTSuV1 in infected pigs. (a) Detection of TTSuV1 DNA. Genomic copy numbers per gram of TBLN and spleen were evaluated by a TTSuV1-specific qPCR assay. *X*-axis: DPI. Left, TBLN; right, spleen. *Y*-axis: Log_10_ TTSuV1 genomic copy numbers per gram of tissue. Blue, TTSuV1 group; Pink, TTSuV1+SIV-infected pigs. *P≤0.05 when compared to the uninfected control group, $*P*≤0.05 when compared to the DPI 17 TTSuV1 group, Student’s t-test. (b,c and d) Representative images of TTSuV1 isolated from lung tissue. Green nuclear fluorescence is characteristic of TTSuV1 viral replication. Blue, DAPI-stained nuclei. (b) TTSuV1 only group. (c) PBS group. (d) TTSuV1+SIV group.

To assess viral replication, a TTSuV1-specific qPCR assay was carried out as described before [17,19], with some modifications. DNA was extracted from tracheobronchial lymph nodes (TBLN) and spleen collected on DPI 22 and DPI 35 from euthanized pigs. Lymph nodes and spleen DNA were extracted using the NucleoSpin^®^ VET (Takara Bio, San Jose, CA) manufacturer’s protocol. The qPCR reaction was set up with the QuantiFast Probe PCR Mix (QIAGEN, Valencia, CA) and primers 5′ CTT GAC CGA CGA ATG GAC C 3′ and 5′ GCT TTT TAT TGA GGC ATC 3′ and probe 5′ d FAM-TAA CCC TCT CCT CGG TCT CGA-BHQ 3′. A no-template control and plasmid positive control were included in every run [17]. Viral copy numbers per millilitre were calculated using the standard curve generated from the positive control plasmid encoding the TTSuV1 genome. The assay had a detection limit of 200 copies per millilitre serum and 143 copies per millilitre of tissue (Fig. 3a).

### Isolation of TTSuV1 from lung and spleen lysates

To verify that a replicative virus was produced by experimental infection of pigs with the rescued TTSuV1, pooled spleen and lung lysates obtained from 50 mg of tissue were filtered with a 0.2-µm filter, freeze-thawed at −80 °C three times and centrifuged at 2,500 r.p.m. for 7 min. The lysates were layered onto 50% confluent pig kidney cells (PK-15, CCL3-ATCC, Manassas, VA) and incubated at 37 °C for 72 h. As TTVs are not cytolytic, replicating viruses were visualized by an IFA as described above (Fig. 3b, c).

### Effects of TTSuV1 infection on lymphocyte function

**Fig. 4.**
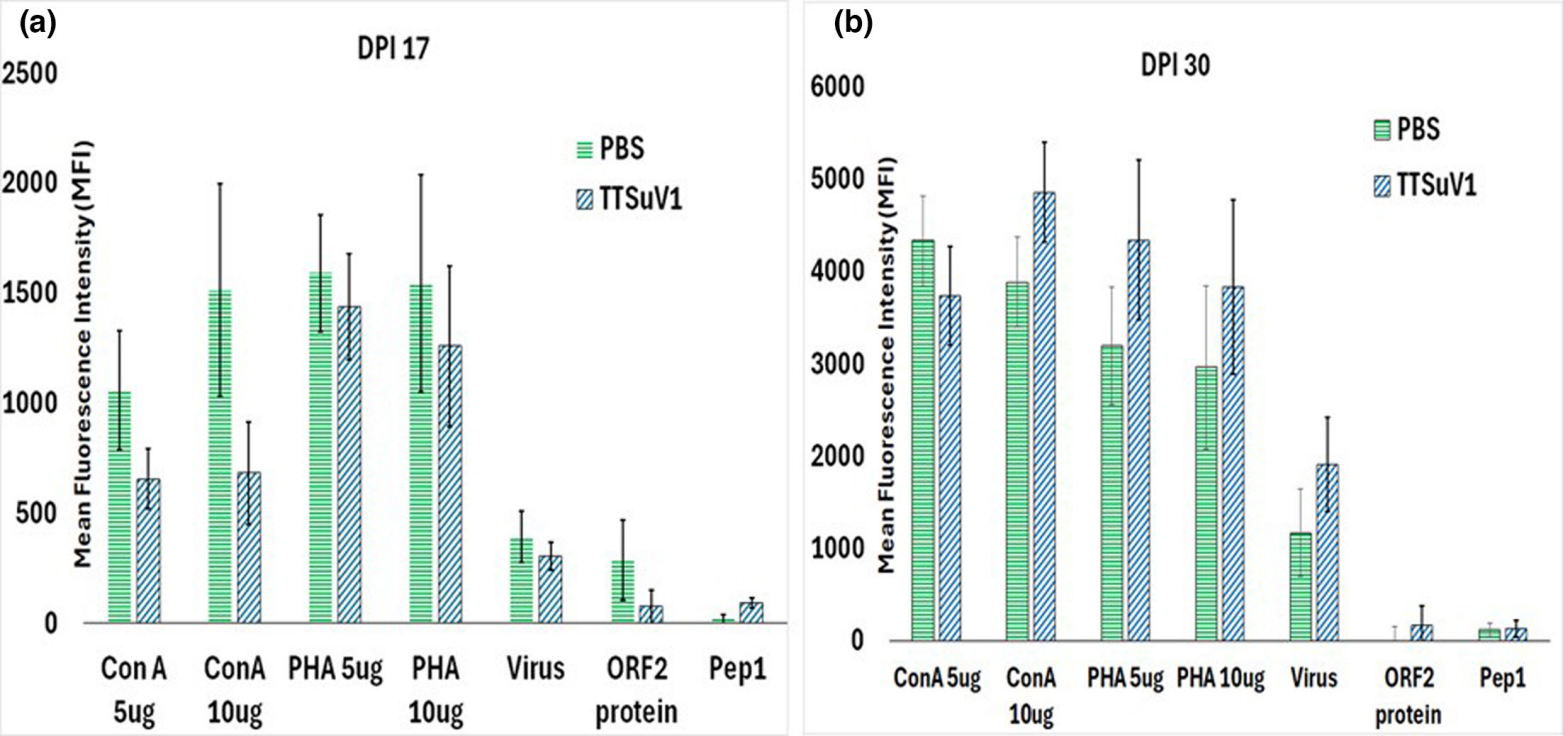
Effect of TTSuV1 infection on mitogenic and TTSuV1-specific recall responses. The ability of PBMCs from the TTSuV1-infected pigs to respond to mitogenic or TTSuV1 antigen stimulation was evaluated by a lymphocyte proliferation assay. (a) DPI 17. (b) DPI 30. *X*-axis: treatments. *Y*-axis: mean fluorescence intensity (MFI). Green, uninfected pigs; blue, TTSuV1-infected pigs. *P≤0.05 when compared to the uninfected control group at the specified time point.

To determine whether TTSuV1 infection affected lymphocyte proliferation responses to mitogens [20] and recall responses to TTSuV1 viral antigens, PBMCs were separated from whole blood collected at DPI 17 and DPI 30 from pigs infected with TTSuV1 alone and the uninfected control pigs. PBMCs were isolated by diluting whole blood 1 : 1 with PBS. RBCs were lysed with ammonium–chloride–potassium lysis buffer (Cat. No. 10-548E, Lonza) for 20 min at RT in the dark. Cells were separated by Ficoll gradient centrifugation. Separated PBMCs were resuspended at 1×10^6^ cells ml^−1^ with 1X RPMI-1640 containing 10% FBS. Cells were stimulated with concanavalin-A (ConA) or phytohaemagglutinin at 5 or 10 µg ml^−1^. Recall responses were assessed with TTSuV1-specific antigens, which included heat-inactivated TTSuV1 virus at 1×10^5^ TCID_50_ ml^−1^, recombinant ORF2 protein [20,23] and a peptide encoding the first 117 amino acids of TTSuV1 ORF1, designated as peptide 1 at a concentration of 50 mg ml^−1^. Assays were run in triplicate and included media-only control wells to obtain baseline data. The proliferative responses were measured using the Alamar Blue reagent (AbD Serotec/Bio-Rad, Raleigh, NC) [30]. Plates were read after 6-h incubation at 37 °C at 570–600 nm wavelength. The mean fluorescence intensity values for DPI 17 and 30 are represented in Fig. 4(a, b).

### Flow cytometry of PBMCs

**Fig. 5.**
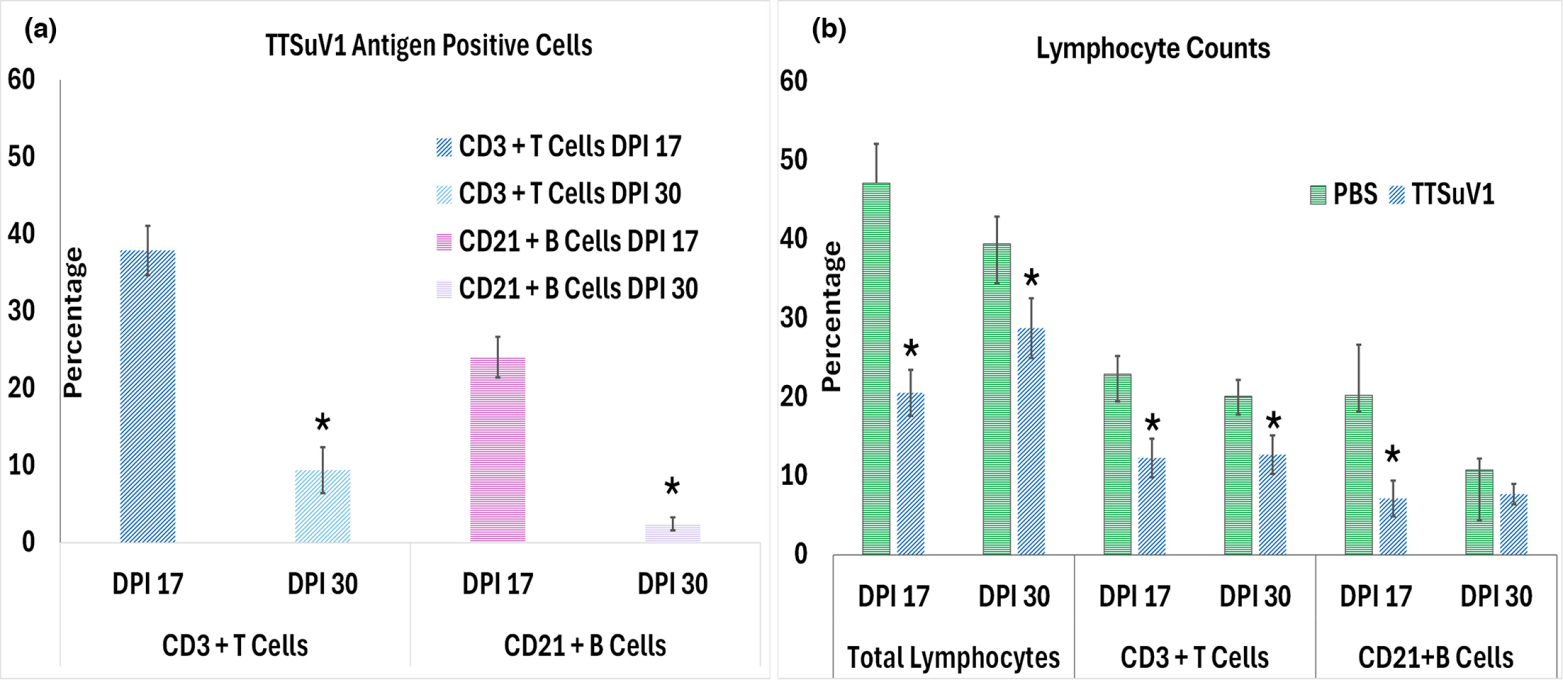
Effect of TTSuV1 infection on lymphocytes. (a) Percentages of TTSuV1-infected cells. *X*-axis: cell types and DPI. *Y*-axis: percentage of cells positive for TTSuV1 antigen. Left, CD3+T cells. Dark blue, DPI 17; light blue, DPI 30. Right, CD21+B cells. Pink, DPI 17; purple, DPI 30. *P≤0.05 when compared to the DPI 17 time point. (b) Changes in lymphocyte numbers in TTSuV1-infected pigs. *X*-axis: cell types and DPI. *Y*-axis: percentage of cells counted. Green, uninfected pigs; blue, TTSuV1-infected pigs. *P≤0.05 when compared to the uninfected control group at the specified time point. The differences between the two time points were not significant.

Flow cytometry was used to detect the presence of TTSuV1 antigen in PBMCs as evidence of direct infection and to evaluate possible changes to lymphocyte numbers due to TTSuV1 infection. PBMCs separated as described above were reconstituted to 1×10^6^ cells and blocked with Fc Blocker (BUF070A, Biorad USA) to remove Fc-mediated binding. 1×10^6^ PBMCs were mixed with either 0.25 µg of T-cell-specific mouse anti-porcine CD3ε-Alexa Fluor^®^ 488 (SouthernBiotech, USA) or 0.5 µg of mouse B cell-specific anti-porcine CD21-Alexa Fluor^®^ 647 (SouthernBiotech, USA), respectively, and incubated at 4 °C for 30 min. Cells were fixed with 4% paraformaldehyde for 30 min. The presence of intracellular TTSuV1 antigen in T and B cells was detected by intracellular staining with a rabbit polyclonal TTSuV1-specific antibody at 1 : 200 dilution for 45 min at RT, followed by anti-rabbit IgG conjugated to PE 1 : 100 (KPL, Gaithersburg, MD) in a 2% BSA+BD Perm/Wash™ (BD Bioscience, USA) solution. Stained cells were analysed using a high-speed cell sorter (BD Accuri ™C6 Plus Flow Cytometer, BD Bioscience, EU). Data were collected for 100,000 events. Dead cells and debris were excluded. Total lymphocytes were gated using the low FSC and high SSC gate. CD21+B cells were identified within the Alexa Fluor^®^ 647 (FL4) channel, and CD3e+T cells were identified within the Alexa Fluor^®^ 488 (FL1A) channel. Intracellular TTSuV1 was identified by distribution in dot plots and by measuring the fluorescent intensity of IgG-PE (FL2A). Assay validation controls included fluorescent minus one staining, single-stained and unstained controls. The mean values for both time points tested are presented in Fig. 5(a, b).

### Evaluation of TTSuV1-induced pathology

**Fig. 6.**
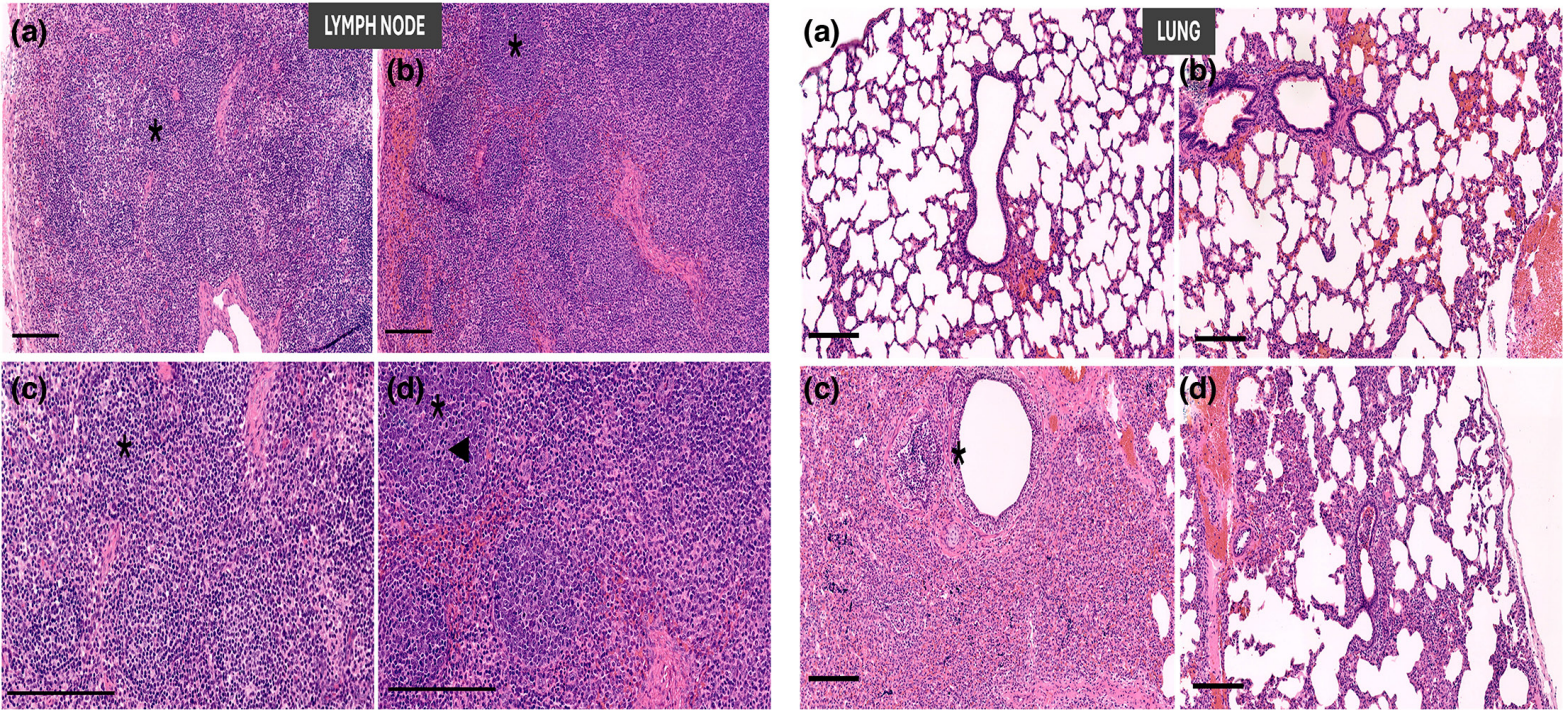
TTSuV1- and SIV-induced tissue lesions. Representative histologic sections stained with haematoxylin and eosin. Top bars, 200 microns; bottom bars, 400 μm. Left panel: lymph node sections from TTSuV1-infected pigs. (a) Uninfected, (b) TTSuV1-infected pig, (c and d) higher magnification of (a) and (b), respectively, demonstrating increased overall cellularity, increased follicle size with prominent germinal centre development (*) and mitotic activity (arrowhead) in (b). Right panel: lung sections from SIV-infected pigs. (a) Uninfected, (b) TTSuV1-infected, (c) infected with SIV only, (d) TTSuV1 and SIV coinfected. Pigs singly infected with SIV (c) had the most severe lesions characterized by consolidation with suppurative bronchopneumonia and bronchiolar epithelial necrosis (*) compared to similar but attenuated changes in pigs infected with SIV only (d).

The major organ systems of experimental animals were evaluated for gross and histological changes by board-certified pathologists who were blinded to the treatments. To evaluate microscopic lesions due to TTSuV1, samples of bone marrow, lungs, liver, spleen, tracheobronchial lymph node, tonsil, mesenteric lymph node, kidney, pancreas, small intestine with Peyer’s patches, lungs, gonads and thymus were collected, routinely processed, sectioned and stained with haematoxylin and eosin. As the primary changes detected in TTSuV1-infected pigs were restricted to lymphoid tissues, a detailed evaluation of splenic tissue was carried out based on Elmore *et al.* [31]. The general scoring scheme for each evaluated criterion was 0, normal; 1, minimal; 2, mild; 3, moderate; 4, marked. As follicular cellularity and germinal centre development are considered important indicators of immunoreactivity, the sum of the scores for the number of follicles, germinal centre development, cellularity of the cords/sinuses and overall cellularity is presented in Table 1 and Fig. 6a. Mitosis in germinal centres was enumerated as the average in a 400× field. Apoptotic changes were evaluated by scoring apoptotic cells and tingible body macrophages. Granulocytes and plasma cells were also evaluated (Table 1).

**Table 1.** TTSuV1 lymphoid activity scores

Treatment	Sum cellularity/germinal centre activity score	Average mitosis score	Average apoptosis score	Average granulocytes score
**DPI 22**				
**Uninfected**	0.00±0.00	2.75±0.50	0.00±0.00	1.50±0.58
**TTSuV1**	2.88±0.49*	4.63±2.20*	1.50±0.18*	1.25±0.46
**SIV+TTSuV1**	3.89±0.42*	6.44±3.05*	2.11±0.55*	0.33±0.50*†
**SIV**	3.67±0.69*	9.17±4.31*†	1.67±0.41*†	0.67±0.52*
**DPI 35**				
**Uninfected**	2.00±0.76	4.50±0.71	3.00±0.58	1.00±0.00
**TTSuV1**	6.82±0.8*	5.25±3.36	2.73±0.00*	1.00±0.00
**SIV+TTSuV1**	7.86±0.37	4.50±1.78*	1.80±0.14 ^†‡^	0.10±0.32^*^^†^
**SIV**	7.20±0.54*	8.40±2.19^*^†	2.40±0.45	0.20±0.45^*^^†^

*P≤0.05 when compared to the uninfected control group, Student’s t-test.

†*P*≤0.05 when compared to the group infected with TTSuV1 only, Student’s t-test.

‡*P*≤0.05 when compared to the group infected with SIV only, Student’s t-test.

### Evaluation of SIV-induced pathology

To evaluate SIV-induced lesions, the total percentage of affected lung parenchyma was scored from 1 to 100%. Individual lung lobes, including the left cranial, left caudal, right cranial, right middle, right caudal and accessory lobes, were sectioned. Microscopic lesion scores were assigned using a previously established rubric [26,32, 33]. Scores of 1 to 4 were assigned based on the percentage of airways affected by lymphocytic cuffing. Severity was also scored from 1 to 4 [26] (Fig. 6b). Tissue sections were stained by an influenza A-specific immunohistochemistry (IHC) assay following the standard operating procedures of the South Dakota State University Veterinary Diagnostic Laboratory. Scores were assigned based on the number of antigen-positive sections. The consolidated gross, microscopic and IHC scores are presented in Table 2.

**Table 2.** Influenza/A/CA/2009 /H1N1 lesion scores

Treatment	Gross lesion	Microscopic lesion	Immunohistochemistry	Total score
**DPI 22**				
**SIV**	19.00±0.05	6.50±0.40	4.83±1.16	30.33±1.22
**SIV+TTSuV1**	16.00±0.11	3.00±0.43*	3.60±1.18	22.60±1.26
**DPI 35**				
**SIV**	9.00±0.05	3.00±0.28	3.0±1.41	15.00±1.18
**SIV+TTSuV1**	3.00±0.03*	0.17±0.07*	1.40±1.51	4.57±1.51

*P≤0.05 when compared to the uninfected control group, Student’s t-test.

Note: The uninfected control pigs and pigs infected with TTSuV1 only did not show any notable gross or microscopic lung lesions.

### Detection and quantification of SIV

#### Quantification of SIV in nasal swabs

**Fig. 7.**
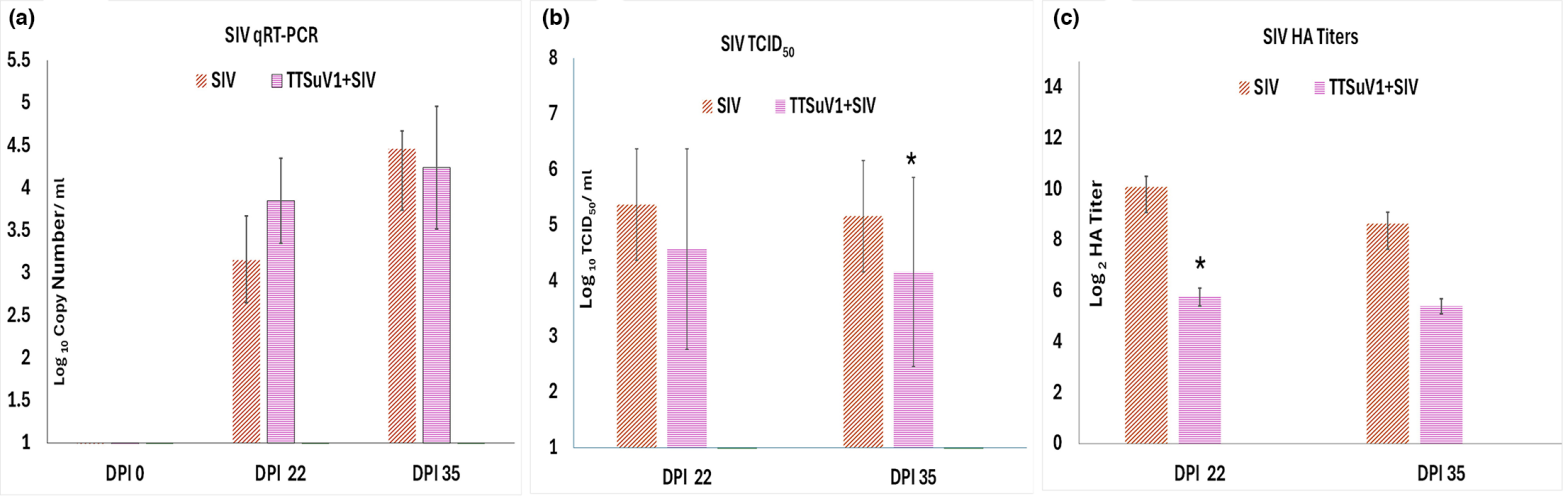
Detection and quantification of SIV. (a) SIV RNA quantification in nasal swabs by quantitative real-time PCR. SIV RNA copy numbers in nasal swab samples were quantified by a SIV-specific qPCR. *X*-axis: DPI. *Y*-axis: Log _10_ copy numbers per millilitre. Brown, pigs infected with SIV only; pink, pigs coinfected with SIV and TTSuV1. Significant differences were not detected between groups at the specified time point. (b) Quantification of SIV isolated from lung lysates by the TCID_50_ assay: lung lysates from pigs infected with PBS, TTSuV1 alone or TTSuV1 plus SIV were used to isolate SIV on pre-formed MDCK monolayers. *X*-axis: DPI. *Y*-axis: mean Log_10_TCID_50_ ml^−1^. Brown, pigs infected with SIV only; pink, pigs coinfected with SIV and TTSuV1. *P≤0.05 when compared to the SIV only control group at the specified time point. (c) Quantification of isolated SIV cultures by a haemagglutination assay. *X*-axis: DPI. *Y*-axis: mean log _2_ HA titre. Brown, pigs infected with SIV only; pink, pigs coinfected with SIV and TTSuV1. *P≤0.05 when compared to the SIV-only control group at the specified time point.

Replication of SIV was evaluated using nasal swabs and a SIV-specific quantitative real-time PCR (Path-ID RT-PCR Kit, Thermo Fisher, USA) targeting the matrix gene. The assay was carried out following the manufacturer’s instructions and as per the protocols of the N. Dakota State University Veterinary Diagnostic Laboratory [26]. A serial log dilution of an SIV virus culture previously quantified by the TCID_50_ method was used to prepare a standard curve and derive copy number values (Fig. 7a).

### Isolation and quantification of SIV from lung lysates

Flash-frozen lung tissue was cut into 500 mg pieces and finely macerated through a 0.70-µm nylon filter (Corning Cell Strainer, Sigma-Aldrich, St. Louis, MO, USA) until a homogenized lysate was obtained. The lysate was centrifuged at 2,500 r.p.m. for 7 min to remove debris. The supernatant was overlaid on pre-formed MDCK monolayers and incubated in DMEM with 1% penicillin–streptomycin, 2% FBS and 1 μg ml^−1^ of TPCK trypsin at 37 °C in 5% CO_2_ incubator for 72 h. Plates were freeze-thawed three times, and the contents were collected to quantify the isolated SIV by the TCID_50_ assay in duplicate. Media control and positive SIV control were maintained for each assay (Fig. 7b).

### Haemagglutination assay of lung lysates

To further verify the viral loads in lung lysate isolates by a second method, a haemagglutination assay (HA) was performed according to the standard protocols of the World Health Organization [34]. Briefly, the lysate was treated with receptor-destroying enzyme (RDE) (1 : 3 dilution) (Denka Seiken, USA) to avoid nonspecific haemagglutination and incubated at 56 °C for 30 min for RDE inactivation. Serial twofold dilutions of virus cultures were performed in 96-well U-bottom plates. Fifty microlitres of 0.5% chicken RBCs (Lampire Biological Laboratories, USA) was added to each well. Plates were incubated at room temperature for 15 min. The haemagglutination titration endpoint was defined by the highest dilution of virus that causes complete haemagglutination (Fig. 7c).

### Statistical analysis

Microsoft Excel was used for all data analysis. Data were analysed for normality, and statistical significance was set at *P*<0.05. All quantitative data were analysed by the parametric Student’s t-test. The figures and graphs include mean or consolidated adjusted values with standard deviations for all readouts.

## Results

### TTSuV1 antibody responses

Antibody responses to TTSuV1 infection were evaluated by a TTSuV1 ORF2-specific ELISA and virus neutralization assays [18,19]. Seroconversion to TTSuV1 was detected by DPI 17 in both the single infection group and the TTSuV1 and SIV dual infection group and continued to increase until the end of the study. The differences in antibody levels between the single and dual infection groups were not statistically significant at any of the time points tested (Fig. 2a). The pigs that were administered PBS remained negative throughout the study. By DPI 17, virus-neutralizing responses in TTSuV1-infected pigs were at 41.35%, and the magnitude of the response increased significantly to 61.48% at DPI 35. The differences between the uninfected pigs and infected pigs were statistically significant at both DPI 17 and DPI 35 time points (Fig. 2b).

### SIV antibody responses

IgG and IgM responses to SIV were evaluated with an H1N1 N protein-specific ELISA. As expected, SIV-specific antibody responses remained low for the short 6-day post-observation period after infection with SIV. The IgM responses were slightly higher than the IgG responses by day six post-infection. The values of the SIV-infected groups differed significantly from the PBS and TTSuV control groups both at DPI 22 and 35. There were no significant differences between the SIV-only group and the SIV+TTSuV1 group, except at DPI 17 for the IgM ELISA (Fig. 2c, d).

### Detection of TTSuV1 DNA in infected pigs

To evaluate TTSuV1 replication in infected pigs, the presence of TTSuV1 DNA in the spleen and tracheobronchial lymph nodes was evaluated using a TTSuV1-specific qPCR. Viral DNA was detected at statistically significant levels at DPI 22 and 35 time points, in both spleen and lymph node tissues. At DPI 22, the TTSuV1 plus SIV coinfected pigs had higher levels of DNA than pigs singly infected with TTSuV1. Copy numbers declined slightly in both tissues by DPI 35, suggesting the beginning of viral clearance, but the differences were not statistically significant (Fig. 3a).

### Isolation of TTSuV1 from infected pigs

To ensure that the detection of TTSuV1 DNA corresponded to productive viral replication, pooled lung and spleen lysates were used to isolate replicative TTSuV1 from the study piglets. Apple green nuclear fluorescence indicative of TTSuV1 replication was detected by a TTSuV1-specific IFA from both TTSuV1 singly infected pigs as well as TTSuV1 and SIV coinfected pigs at both time points. The virus isolations from the PBS group remained negative. The results confirmed that viable TTSuV1 was indeed produced by experimental infection with the recombinant rescued TTSuV1 culture used for the experimental infection of the piglets (Fig. 3b, c and d).

### Lymphocyte proliferation responses

To evaluate the effects of TTSuV1 on lymphocyte function, mitogenic and virus-specific recall responses in TTSuV1-infected pigs were measured by a lymphocyte proliferation assay. At DPI 17, the proliferation responses of PBMCs from TTSuV1-infected pigs to both mitogens and TTSuV1-specific antigens were diminished compared to the uninfected controls, especially for ConA. Virus-specific recall responses were extremely low or absent at DPI 17. The diminished response to mitogenic stimuli in TTSuV1-infected pigs was reversed by DPI 30. Recall responses to the inactivated virus preparation and the ORF2 recombinant antigen increased slightly. The differences between the PBS group and the TTSuV1-infected group were not statistically significant for all treatments at both time points (Fig. 4a, b).

### Effect of TTSuV1 infection on lymphocytes

Detection of intracellular TTSuV1 antigen in lymphocytes by flow cytometry as an indicator of direct infection revealed that 38% and 24% of T cells and B cells, respectively, contained TTSuV1 antigen at DPI 17. The percentage of infected cells declined to 24.4% and 2.4% T and B cells, respectively, by DPI 30, indicating that cell-associated TTSuV1 was being cleared at the later time point (Fig. 5a). Evaluation of whether TTSuV1 infection resulted in changes to lymphocyte numbers by flow cytometry showed that TTSuV1 infection resulted in a significant reduction in the number of total lymphocytes, CD3+T cells and CD21+B cells in TTSuV1-infected pigs when compared to uninfected pigs, at both time points tested. The reduction in lymphocyte numbers was greater at DPI 17 compared to DPI 30, but the differences between the two time points were not statistically significant (Fig. 5b).

### TTSuV1 pathology

The TTSuV1-infected pigs did not show significant gross pathological changes in organs and tissues during necropsy. Although tissues examined microscopically did not show significant changes, changes were noted in lymphoid tissues. Severe lymphoid depletion was noted in one singly infected pig at each time point by a detailed examination of the spleen [31]. Compared to uninfected pigs, cellular/germinal centre activity and mitotic activity were higher in all infected pigs at both time points. However, coinfected pigs had significantly lower mitotic numbers, indicating that the presence of TTSuV1 reduced the mitotic activity of SIV. Apoptotic activity was significantly increased in all infected groups at DPI 22 but not at DPI 35. SIV infection significantly reduced the scores for granulocytes in both the single and dual infection groups. Significant numbers of plasma cells were not detected. Overall, single TTSuV1 infection resulted in increased cellularity, germinal centre activity and apoptosis at DPI 22 (Table 1). Mitotic numbers were significantly lower in coinfected animals compared to animals infected with SIV alone (Table 1, Fig. 6A). While statistical differences were observed across groups in the apoptosis and granulocyte scores, they are not depicted in Fig. 6A, as they were evaluated across biological replicates and are difficult to visually appreciate in individual photomicrographs.

### SIV pathology

While it was expected that TTSuV1 plus SIV coinfected pigs would experience more severe infection than pigs infected with SIV alone, the gross, microscopic and immunohistochemistry scores were consistently lower in pigs administered TTSuV1 prior to SIV infection compared to singly infected pigs at both DPI 22 and DPI 35. The differences in gross lesions were statistically significant at DPI 35, while the microscopic lesions showed significant differences at both the DPI 22 and 35 time points (Table 2, Fig. 6b). The overall SIV lesion scores were higher in pigs at DPI 22, when compared to pigs at DPI 35. As expected, the uninfected control pigs did not show any remarkable changes.

### Clinical signs

As expected, overt clinical signs were not observed in the TTSuV1-infected pigs throughout the study. As is common in experimental models, the pigs infected with SIV did not experience severe respiratory distress or show signs of SIV infection [26,35]. None of the study pigs lost more than 20% of body weight during the study and overall remained in good condition until euthanasia.

### Detection and quantification of SIV

To evaluate if TTSuV1 coinfection influenced SIV replication, nasal swabs were subjected to SIV-specific real-time qPCR. All SIV-infected pigs were SIV RNA positive at both points tested, while the PBS controls remained negative. The difference between the single and dual infection groups was not statistically significant at DPI 22 and 35 (Fig. 7a). To ensure that experimental SIV infection resulted in SIV replication in pigs, SIV was isolated from lung tissues, and the TCID_50_ assay was used to quantify SIV loads in the lung tissue lysates. The HA assay was used to verify the TCID_50_ data by a second method. Viral loads in the isolated virus cultures measured by both the TCID_50_ and HA assays showed that the TTSuV1 and SIV coinfected pigs had lower SIV loads at both DPI 22 and 35 compared to pigs singly infected with SIV. The differences were statistically significant at DPI 35 time point by the TCID_50_ assay (Fig. 7b) and at DPI 22 by the HA assay (Fig. 7c). The control pigs in the PBS group remained SIV negative, as expected.

## Discussion

Similar to the bacterial microbiome, the human virome comprises ~10¹³ viral particles per individual, exhibits substantial heterogeneity and plays a critical role in modulating health and disease outcomes [36]. Among the most ubiquitously detected viral families in metagenomic studies are the *Anelloviridae* [37]. The principal genus within this family, TTV, is strongly associated with immunosuppression and a range of disease conditions, including autoimmune disorders, hepatitis and respiratory infections, and is suspected to act as a potentiating agent for coinfecting pathogens [4]. Despite their prevalence, progress in understanding TTV biology has been limited by the absence of robust *in vitro* and *in vivo* experimental systems. Consequently, the molecular mechanisms underlying their capacity to establish lifelong infections and modulate host–virus or virus–virus interactions remain poorly defined [4,16]. To address these knowledge gaps, the present study aimed to expand experimental models for investigating the infectivity of recombinant TTSuV1 in conventional piglets [18] and to assess the impact of TTSuV1 coinfection on the pathogenesis of SIV.

Previously published studies investigating experimental infection of pigs with TTSuVs utilized bone marrow or liver lysates derived from naturally infected animals as inocula [9,11]. A major limitation of this approach is the potential presence of extraneous infectious agents within the inoculum, which complicates the interpretation of experimental outcomes. Huang *et al*. [38] demonstrated that direct administration of cloned TTSuV DNA via intramuscular or intra-lymphoid routes could establish infection in piglets. However, because high-titre propagation of TTSuVs *in vitro* remains technically challenging, that study relied on the use of plasmid DNA for inoculation rather than rescued infectious virus culture. This approach complicates differentiation between antibody responses to replication-competent virus and those elicited by transient expression of viral proteins from the inoculated DNA, as well as distinguishing residual input DNA from the inoculum from replicating viral genomes by qPCR [38]. We have previously shown that supplementing cell cultures with replicase proteins derived from circoviruses in trans can overcome the bottleneck associated with producing sufficiently high titres of TTSuV1 *in vitro* [17]. Consequently, the present study is unique in using recombinant, cell-culture-rescued TTSuV1 as the inoculum for experimental infection. Furthermore, whereas prior infection studies were conducted in gnotobiotic pigs, which are costly and labour-intensive to maintain, this work establishes the snatch-farrowed piglet as a practical and biologically relevant alternative model for controlled TTV studies.

Data from several field studies demonstrate a lack of correlation between antibody responses to TTSuVs, disease manifestation and viral load [39,41]. Although limited information exists regarding serological profiles following experimental TTSuV infections in pigs, our findings revealed that only ~60% virus neutralization was achieved by 30 DPI, and neutralizing antibody titres did not correspond with the strong binding antibody responses detected at DPI 17 (Fig. 2a, b). The observed virus neutralization response resulted in only a ~2 log reduction in viral load by the end of the study (Fig. 2b), suggesting that TTSuV1 may evade host immunity through mechanisms that subvert effective antibody-mediated neutralization. As SIV causes acute infections with peak viral loads detected by day 5–7 post-infection, the experimental pigs were euthanized at 5 days post-SIV infection. This time frame was too short for evaluating SIV neutralizing antibody responses, which often peak between 14 and 21 dpi [42]. Therefore, anti-SIV neutralizing antibody responses were not measured.

TTV titres are found to be higher in whole blood or cell-associated fractions than in serum [43,44]. In this study, TTSuV1 DNA was readily detected in spleen and lymph node tissues (Fig. 3a). A possible cell-associated replication pattern, together with prior observations that older pigs exhibit higher TTSuV DNA loads than younger animals [41], suggests that delayed viremia may represent a unique feature of TTV infection that facilitates antibody escape. Although the study was concluded at DPI 35 and did not capture longer-term infection dynamics, the successful re-isolation of TTSuV1 from tissues of infected pigs (Fig. 3b,c), along with the detection of TTSuV1 antigen within lymphocytes by flow cytometry (Fig. 5a), provides confirmatory evidence that the rescued recombinant TTSuV1 was infectious and capable of productive replication *in vivo.*

Previous studies show that coinfection of pigs with TTSuV1 and either PCV2 or PRRSV [9,10] exacerbated disease severity and led to the manifestation of PCV-associated disease and porcine dermatitis and nephropathy syndrome, respectively. Therefore, the finding that prior infection with TTSuV1 resulted in reduced SIV replication in the lungs in this study was unexpected (Fig. 6b, c). The presence of other unintended TTSuV1 strains in the experimental system could lead to differential observations noted, but it is highly unlikely due to the snatch farrowing method followed. Moreover, unpublished data from subsequent RNA-Seq analysis of tissue from the experimental piglets were negative for transcripts that matched other TTVs. Experimental infection of conventional pigs with the 2009/H1N1 SIV strain typically produces only mild clinical disease [26,35]. Further, to maintain virulence, the 2009/H1N1 SIV strain originally obtained as a cell passage 1 from CDC was back-passaged by re-isolation from pig lung tissue following a prior challenge study [25]. The back-passaged and re-isolated virus stocks were expanded and stored at −80 °C until further use. The third passage after re-isolation was used in this study. Accordingly, although alterations in viral replication patterns were observed, no significant changes in clinical signs were detected. Notably, reduced SIV replication in the coinfected group corresponded with lower gross pathological, microscopic and immunohistochemistry scores relative to pigs infected with SIV alone (Table 2, Fig. 6b). While the use of alternate or more virulent SIV strains could alter study outcomes, this study was limited in the number of viral strains tested due to logistical reasons.

There is strong clinical and experimental evidence that TTVs are lymphotropic and engage in complex interactions with the host immune system [22,45, 46]. Consistent with previous findings, we observed a loss of lymphocyte responsiveness to mitogens in TTSuV1-infected piglets (Fig. 4a, b), a phenomenon that we and others have previously reported in TTSuV1-transfected swine PBMCs [20,47]. A particularly striking finding in this study was the absence of detectable recall responses to TTSuV1 antigens (Fig. 4a, b). Although the specific characteristics of TTSuV1-induced memory responses were not examined in detail, these results suggest that immune memory subversion, together with the high genetic diversity characteristic of TTVs [2], may represent a key mechanism enabling persistent and chronic infection. Furthermore, direct infection and depletion of lymphocytes by TTSuV1 (Fig. 5a) likely contribute to early immune suppression, facilitating viral establishment within the host. A limitation of this study is that a more detailed analysis of cytokine responses and other immunomodulatory mechanisms was not undertaken [48]. It is well established that TTV loads increase in immunosuppressed individuals with T-cell depletion [49], implicating T cells as critical mediators of viral clearance. Infection of T cells by TTSuV1 may, therefore, significantly impair their antiviral function (Fig. 5a). Supporting this hypothesis, a recent study demonstrated that TTV-specific CD8^+^ T cells exhibit an exhausted phenotype characterized by diminished IFN-*γ* production and high expression of the inhibitory receptor NKG2A. TTV-derived peptides were shown to bind NKG2A and suppress both CD8^+^ T-cell and NK-cell activity [47], providing compelling mechanistic support for the observations reported here.

A range of pathological alterations, including thymic atrophy, interstitial pneumonia and hepatic and renal lesions, have been reported in gnotobiotic pigs experimentally infected with TTSuV1, while naturally infected pigs exhibited mild lymphoid hyperplasia, lymphoid depletion and granulomatous inflammation [10,50]. In the present study, which utilized a conventional snatch-farrowed pig model infected with recombinant TTSuV1, overt pathological changes were not observed. However, the presence of well-developed germinal centres in bronchial and mesenteric lymph nodes was consistent with findings reported by Krakowka *et al*. [10] during experimental TTSuV1 infection (Table 1). Similarly, the observed B-cell hyperplasia aligned with lesions described in naturally infected pigs [50]. Follicular cellularity and germinal centre formation are sensitive indicators of immune reactivity [31]. The increased cellularity, germinal centre activity and mitotic figures observed in this study suggest that TTSuV1 infection elicited mild to moderate immune activation (Table 1, Fig. 6a), consistent with the induction of binding antibody responses (Fig. 2a). Viruses frequently manipulate the host cell cycle by disrupting regulatory checkpoints to facilitate replication. For instance, the Zika virus induces mitotic abnormalities and apoptosis in neural progenitor cells [28]. Previous studies have proposed that TTVs can induce apoptosis [51,52], a finding supported by the microscopic lesions observed in this study (Table 1). Furthermore, the reduced granulocyte scores recorded in SIV-infected pigs were consistent with the granulocyte depletion previously reported in infections with the 2009 pandemic H1N1 strain [53].

The unexpected observation that prior infection with TTSuV1 conferred protection against SIV-induced pulmonary pathology (Table 2) supports previous reports suggesting that interactions between coinfecting viruses can yield complex and sometimes paradoxical outcomes [54]. This finding underscores the persistent challenge of accurately reproducing multifactorial field conditions under controlled experimental settings. Further, the timing or order of infection of TTSuV1 and SIV, duration of the study and sample collection times are other factors that could influence study outcomes. For example, reducing the time interval between TTSuV1 and SIV infection may have reduced the mitigating effect of TTSuV1 due to the time required for TTSuV1 to establish infection [18]. A limitation of this study is that an exhaustive exploration of other possible study designs was not conducted. Viral coinfections are known to either enhance or inhibit each other’s replication and pathogenesis through a variety of mechanisms. These may include modulation of antiviral immune responses, alteration of inflammatory signalling, interference with antiviral RNA interference pathways, differential activation of host immune mediators, expression of trans-acting viral proteins or proteases and competition for cellular receptors or replication factors [55,56]. In previous *in vitro* studies, we reported that infection of 3D4/31 macrophages cells with TTSuV1 or over-expression of TTSuV1 ORF1 protein resulted in upregulation of immune regulatory molecules like SOCS-1, PD-L1 and IL-10 [23], which could potentially dampen the pro-inflammatory responses involving chemokines, IL-6, TNF-*α*, IL-8 or IL-1*β* commonly associated with SIV infections [57,58]. Although elucidating the precise molecular pathways underlying the TTSuV1–SIV interaction was beyond the scope of this study, future investigations will aim to address these mechanisms in detail. Importantly, this observation may have broader implications for understanding the potential role of early-life TTV colonization in shaping immune development, mitigating excessive inflammatory responses and establishing homeostatic balance within the host virome–microbiome ecosystem.

In summary, the recombinant TTSuV1 derived from the infectious clone was demonstrated to be infective in pigs. Infection with TTSuV1 resulted in alterations in lymphocyte numbers and function and, unexpectedly, conferred protection against SIV infection in coinfected animals. The establishment of a less technically demanding snatch-farrowed pig model, combined with the availability of pure recombinant TTSuV1 cultures, provides a valuable platform for advancing research on TTSuVs and for more precisely elucidating the mechanisms governing TTV interactions with other coinfecting agents.

## Abbreviations

ConA: concanavalin-A
DPI: day post-infection
ELISA: enzyme-linked immunosorbent assay
HA: haemagglutination assay
IFA: immunofluorescence assay
IHC: influenza A-specific immunohistochemistry
MDCK: Madin–Darby Canine Kidney
PBMCs: peripheral blood mononuclear cells
PCV: porcine circovirus
PCV2: porcine circovirus type 2
PRRSV: porcine reproductive and respiratory syndrome virus
qPCR: quantitative PCR
RDE: receptor-destroying enzyme
RT: room temperature
SIV: swine influenza virus
TBLN: tracheobronchial lymph nodes
TTSuV1: Torque teno sus virus 1
TTSuVs: Torque teno sus viruses
TTVs: Torque teno viruses
